# Characterizing the performance of an antibiotic resistance prediction tool, gnomonicus, using a diverse test set of 2,663 Mycobacterium tuberculosis samples

**DOI:** 10.1099/mgen.0.001592

**Published:** 2025-12-15

**Authors:** Jeremy Westhead, Catriona S. Baker, Marc Brouard, Matthew Colpus, Bede Constantinides, Alexandra Hall, Jeff Knaggs, Marcela Lopes Alves, Ruan Spies, Hieu Thai, Sarah Surrall, Kumeren Govender, Timothy E.A. Peto, Derrick W. Crook, Shaheed V. Omar, Robert Turner, Philip W. Fowler

**Affiliations:** 1 Nuffield Department of Medicine, University of Oxford, Oxford, UK; 2 Ellison Institute of Technology, Oxford Ltd, Oxford, UK; 3 National Institute of Health Research Oxford Biomedical Research Centre, University of Oxford, Oxford, UK; 4 Health Protection Research Unit in Healthcare Associated Infections and Antimicrobial Resistance, John Radcliffe Hospital, Oxford, UK; 5 Centre for Tuberculosis, National & WHO Supranational TB Reference Laboratory, National, Institute for Communicable Diseases, a division of the National Health Laboratory Service, Johannesburg, South Africa

**Keywords:** antimicrobial resistance, diagnostics, tuberculosis (TB), whole-genome sequencing (WGS)

## Abstract

Tuberculosis remains a global health problem. Making it easier and quicker to identify which antibiotics an infection is likely to be susceptible to will be a key part of the solution. Whilst whole-genome sequencing offers many advantages, the processing of the genetic reads to produce the relevant public health and clinical information is, surprisingly, often the responsibility of the end user, which inhibits uptake. Here, we characterize how well a freely available tool we have developed, gnomonicus, predicts the antibiotic resistance profile of a sample (given its variant call file) using our implementation of the second edition of the World Health Organization (WHO) catalogue of resistance-associated variants (WHOv2). To facilitate this, we have constructed a diverse test set of 2,663 publicly available *Mycobacterium tuberculosis* samples, which have both genetic and drug susceptibility testing (DST) data. We have chosen to apply the catalogue such that our tool will return a result of (i) Fail if there are insufficient reads at a genetic locus associated with resistance, (ii) Unknown if a genetic variant in a resistance gene not listed in the catalogue is encountered and (iii) Resistant if three or more short-reads support the presence of a resistance-associated variant. The last step increases the sensitivity for all 15 antibiotics but only reaches significance in a few in our test set. Comparing our results with those of TB-Profiler, an existing tool, highlights the different design choices and demonstrates that the performance of both tools on our diverse test set is comparable. By only considering high-confidence DST results, we show that gnomonicus, in combination with our translation of WHOv2, achieves sensitivities and specificities in excess of 95% for both isoniazid and rifampicin.

**Impact Statement**

Whole-genome sequencing of clinical samples taken from patients with tuberculosis is a potentially fast and accurate method for determining to which antibiotics the infection will be susceptible. Two barriers need to be overcome: the first, which is knowing which mutations are associated with resistance (or not) to a range of antibiotics, is well on the way to being solved thanks to the efforts of the World Health Organization (WHO), who have published extensive catalogues containing lists of such mutations. The second barrier is that the processing of the raw genetic files remains a largely manual process overseen by bioinformaticians. Here, we describe gnomonicus, our open-source antimicrobial resistance (AMR) prediction tool, and report the performance of our translation of the second edition of the WHO catalogue using a carefully designed, publicly available dataset of 2,663 *Mycobacterium tuberculosis* samples. We hope that not only will this tool be useful but also that this dataset will be used by other researchers to facilitate comparisons between pipelines, approaches and tools.

## Data Summary

The attendant GitHub repository [1] allows gnomonicus to be rerun on all 2,663 samples in the diverse test set; it therefore includes instructions, the necessary input files (all the variant call files, the version of the WHOv2 catalogue and a link to the H37Rv GenBank file used in this study) and also the output JavaScript Object Notation (JSON) files. The European Nucleotide Archive (ENA) accession numbers for all 2,663 samples (including a bash script to download them) and their corresponding phenotypic drug susceptibility testing results are included with the intention that people can either reproduce our results, or use the same dataset for other analyses. The JSON files output by TB-Profiler have also been added for comparison. The repository contains a series of Jupyter notebooks containing Python 3 code that allows the user to discover and parse the output JSON files from either tool and save the results as data tables. Other notebooks allow the user to reproduce all the analysis underlying this work, including reproducing the figures and many of the tables.

## Introduction

In 2023, just under 11 million people became ill with tuberculosis (TB) worldwide, and 1.25 million died [2]. The aetiological agent, *Mycobacterium tuberculosis*, requires treatment with multiple antibiotics. Drug-susceptible *M. tuberculosis* is routinely treated with four antibiotics: rifampicin, isoniazid, pyrazinamide and ethambutol. Like other bacterial pathogens, resistance to all antibiotics has now been observed, and multi-drug-resistant (MDR) TB is defined as infections resistant to both rifampicin and isoniazid – these require alternative treatment, such as the BPaLM (bedaquiline, pretonamid, linezolid and moxifloxacin) regimen that was recommended by the World Health Organization (WHO) in 2022 [3]. MDR-TB is increasingly recognized as a global health concern, and consequently rifampicin-resistant *M. tuberculosis* was added to the WHO Bacterial Priority Pathogens List in 2024 [4]. Positive identification of *M. tuberculosis* complex in a clinical sample and subsequent phenotypic drug susceptibility testing (pDST) are important steps in treating this disease.

Due to the slow growth rate of *M. tuberculosis,* culture-based pDST takes weeks; whole-genome sequencing (WGS) is an attractive alternative since it is faster, potentially more accurate and also yields epidemiological information. Over the last 8 years [5], many high-income countries have adopted WGS for *M. tuberculosis*. Our ability to accurately predict the antibiogram of a sample from its genetics has been boosted in recent years by the systematic sequencing and drug susceptibility testing of large numbers of clinical samples by projects such as the Comprehensive Resistance Prediction for Tuberculosis: an International Consortium (CRyPTIC). CRyPTIC collected over 15,000 clinical samples via 14 laboratories based in 11 countries [6]. This and other publicly available datasets enabled the WHO to release in 2021 the first catalogue of mutations in *M. tuberculosis* complex associated with drug resistance [7]. A second edition was released in late 2023 [8], which we will call WHOv2. Both editions take the form of a traditional text-based report (which contains some expert rules) and an accompanying detailed Excel spreadsheet (with more rules), with the second edition also including a variant call file. Each edition used a single dataset to both infer the association of genetic variants with antibiotic resistance and estimate the performance of the resulting catalogue. It is challenging to apply either catalogue to the WGS data of a clinical sample, since no computational tool was released with either catalogue, nor was an annotated test dataset released that would have simplified tool development.

In this article, we describe a tool, gnomonicus, we have developed that ingests a resistance catalogue, a sample variant call file and the GenBank file of the reference genome, and outputs the mutations detected and their predicted effects on a range of antibiotics. Since no validation dataset is publicly available, we have also built a diverse test sett of 2,663 *M*. *tuberculosis* samples that have undergone both WGS and pDST. This will enable us to calculate the performance of our implementation of the WHOv2 catalogue and compare it to the stated performance. Finally, we compare our results with those of TB-Profiler [9], which is the best-known third-party tool that performs a similar function.

## Methods

### Sample selection

A total of 11,887 samples were identified from v2.1.2 of the publicly available CRyPTIC dataset [10] that (i) had been whole-genome sequenced using short-read (Illumina) technologies and (ii) had minimum inhibitory concentrations (MICs) to 13 different antibiotics measured using a bespoke 96-well broth microdilution (BMD) plate. Each sample was sequenced and incubated on a BMD plate as described previously [6,11]. In addition to the MICs measured visually by the laboratory scientist, photographs of each plate were image processed [12] and classified by at least 11 volunteers as part of a citizen science project [13]. All MICs where two or three of these independent measurements agreed were annotated as high-confidence MICs – these are assumed to have reduced measurement error. Two plate designs were used (UKMYC5 and UKMYC6), each of which included 13 antibiotics: amikacin, bedaquiline, clofazimine, delamanid, ethambutol, ethionamide, isoniazid, kanamycin, levofloxacin, linezolid, moxifloxacin, rifabutin and rifampicin. All MICs were converted to a binary Resistant/Susceptible result using a set of research epidemiological cutoff values (ECOFF/ECVs) [11]. All 13 antibiotics, except rifabutin, are included in the WHOv2 catalogue. The WHOv2 catalogue also includes three drugs not present on the plates: pyrazinamide, capromycin and streptomycin. We therefore also identified a further 10,606 publicly available samples from the CRyPTIC dataset [10], which had at least one binary phenotype for these drugs measured via the BD BACTEC™ MGIT™ 960 system. In practice, many of these samples will have been tested using both pDST methods; however, to avoid any potential inconsistencies introduced by combining the results from different methods, each sample is associated with only one pDST method.

### Construction of the diverse test set

The diverse test set has two competing aims: (i) to have as close to 50% resistance/50% susceptibility for all drugs to maximize resolution, whilst also (ii) being as small as possible to enable rapid, repeated testing. The difficulty being, of course, that each sample selected has phenotypic information for more than one drug, which makes it difficult to achieve the first criterion. Finally, there is a risk of introducing bias if all samples have some degree of resistance. We therefore arbitrarily decided to create an initial dataset of 1,000 samples with phenotypes for the 13 drugs on the UKMYC plate designs, ensuring that 200 of these were pan-susceptible.

Since the CRyPTIC project collected very few samples resistant to the new and repurposed drugs, we first selected all 284 samples which were assessed as resistant to one or more of bedaquiline (*n*=65), linezolid (117) and delamanid (140). Additional samples were then randomly selected if they (i) were resistant to the remaining drug with the fewest resistant samples, (ii) were not resistant to any drugs that had already reached 50% penetration in the dataset and (iii) were drawn from the remaining 100 samples with the greatest number of high-confidence MICs. This process was repeated until 800 samples had been chosen, whereupon a further 200 pan-susceptible samples were chosen from the 1,000 pan-susceptible samples with the greatest number of high-confidence MICs.

Despite our efforts, there are fewer than 250 resistant samples for bedaquiline, linezolid and delamanid in the UKMYC dataset (Table 1). We therefore repeated the process of iteratively selecting individual samples from the dataset of 10,606 samples with Mycobacterial Growth Indicator Tube (MGIT) pDST data, the main differences being (i) we only considered pyrazinamide, capreomycin, streptomycin, linezolid and bedaquiline and (ii) each sample had pDST results for a variable number of these drugs, and therefore we tried to maximize the number of pDST results per sample. After de-duplication with the UKMYC dataset and verification that all samples had raw genetic (FASTQ) files available in the European Nucleotide Archive (ENA), this led to a dataset of 1,663 samples. The number of pDST results per sample (Table S1, available in the online Supplementary Material) varied between one and four, with two being the most common. The aggregated dataset of 2,663 samples therefore contains at least 250 resistant samples for all drugs (Table 1), except linezolid (*n*=117) and delamanid (140). It is important to note that the process to produce the diverse test set was designed to satisfy the aims stated above, but does not ensure diversity in resistance mechanisms.

**Table 1.** Proportion of resistance by drug in the 1,000 UKMYC samples and 1,663 MGIT samples Due to having the highest prevalence of resistance in the CRyPTIC dataset, only isoniazid reached 50% resistance. A key difference between the two datasets is that each UKMYC sample has an MIC and thence a binary phenotype for 13 drugs, whereas the MGIT samples have between one and four binary phenotypes, with two being the most common (702 samples; Table S1). As each UKMYC plate was read by three independent methods, all MICs where at least two methods agree are assumed to be high confidence; this was not possible for the MGIT samples .

Antibiotic	pDST method	% Resistant	R	S	Total	% High confidence
Bedaquiline	UKMYC	6.5	65	935	1,000	85.1
Linezolid	UKMYC	11.7	117	883	1,000	71.9
Delamanid	UKMYC	14.0	140	860	1,000	74.9
Ethambutol	UKMYC	28.8	288	712	1,000	76.7
Moxifloxacin	UKMYC	28.8	288	712	1,000	73.2
Clofazimine	UKMYC	28.8	288	712	1,000	67.6
Amikacin	UKMYC	28.8	288	712	1,000	82.5
Ethionamide	UKMYC	28.8	288	712	1,000	86.3
Kanamycin	UKMYC	28.9	289	711	1,000	88.0
Levofloxacin	UKMYC	32.3	323	677	1,000	79.0
Rifampicin	UKMYC	47.6	476	524	1,000	88.0
Isoniazid	UKMYC	50.0	500	500	1,000	90.6
Linezolid	MGIT	13.5	105	675	780	–
Streptomycin	MGIT	41.0	252	362	614	–
Capreomycin	MGIT	30.8	256	575	831	–
Pyrazinamide	MGIT	46.4	291	336	627	–
Bedaquiline	MGIT	50.9	394	380	774	–

### Identifying the variants in the diverse test set using EIT GPAS

For simplicity, all samples were processed using the Mycobacterial pipeline deployed in the Ellison Institute of Technology (EIT) Global Pathogen Analysis Service (GPAS; https://gpas.global). The pipeline used is described in more detail in the Supplement. Samples were uploaded using its command-line interface (CLI). This ensured that reads matching the human genome were removed using the hostile [14] algorithm prior to upload. Upon arrival in the cloud, all samples undergo a second round of decontamination with hostile [14], then poor-quality/short reads are discarded before the number of reads belonging to the *Mycobacterium* genus is assessed using Kraken2 (v2.1.3) in conjunction with the Standard Index from June 2023 [15]. If a sample has over 10,000 Mycobacterial (and unclassified) short reads, it is progressed. These reads are then competitively mapped to a curated list of 186 Mycobacterial genomes using minimap2 (v2.24-r1122) [16] to provide fine-grained speciation. Last, in the case of complexes, the reads are then examined by Mykrobe (v0.13.0) [17], which classifies reads down to the level of lineage (in the case of *M. tuberculosis* complex) or subspecies (for *Mycobacterium avium* complex and *Mycobacterium abscessus* complex). Reads are then mapped to version 3 of the *M. tuberculosis* H37Rv reference genome [18,20] (NC 000962.3) using Clockwork (v0.12.3) [21], which, in turn, uses minimap2 [16] to build a pile-up, and then both samtools (v1.15.1) [22] and Cortex [23] to call variants; the former being better at identifying SNPs and the latter insertions and deletions, and Minos [24] adjudicates when there is overlap. Clockwork was used by the CRyPTIC project [6] and also for all variant calling in the first edition of the WHO catalogue of resistance-associated variants (RAVs) [7]. Clockwork calls a genetic variant if it is supported by 90% or more of the reads. The variant call files for all 2,663 samples were downloaded and stored in the attendant GitHub repository [1] for later processing by gnomonicus.

### Translation of the second edition of the WHO catalogue of RAVs in *M. tuberculosis* and resistance prediction

As mentioned, WHOv2 comprises three artefacts; no single artefact contains all the rules in the catalogue, and only the Excel and variant call format (VCF) files are parsable by computer code. The Excel file adopts the Human Genome Variation Society (HGVS) nomenclature for describing genetic variants, but this is unable to encode some of the broader rules found in the catalogue, such as ‘*any frameshift in gene X*’, which is a key component of the new Loss of Function rules. The CRyPTIC project [11] developed a Grammar for Antimicrobial Resistance Catalogues (GARC) specifically for this purpose, and we therefore translated the Excel file into the GARC format [25] that can be read and understood by gnomonicus [26]. A formal definition of the GARC grammar is provided via its Backus–Naur Form in the Supplement. Code changes were required to incorporate the new epistatic rules introduced in WHOv2. Our catalogue maps groups 1 and 2 onto Resistant (R) and groups 4 and 5 onto Susceptible (S), as suggested by the WHO, with Group 3 being labelled Unknown (U).

There are three important differences in our implementation of WHOv2; our catalogue will report any mutation in a gene known to be associated with resistance but not in the catalogue as Unknown. If there are two or fewer reads at a genetic locus known to be associated with resistance, our catalogue will return a result of Fail, since there is not enough information to determine if the sample is resistant or not, and it probably needs re-sequencing. Neither scenario is described by WHOv2, and therefore both would be predicted susceptible without these rules. Finally, we call any RAV listed in the catalogue if it is supported by three or more reads, regardless of how many other (usually WT) reads are also found at that locus. Minor alleles with RAVs are therefore detected; by contrast, 75% of reads were required to support a genetic variant for it to be considered for the WHOv2 catalogue, and there is no guidance on what threshold should be applied when detecting variants listed in WHOv2.

### Predicting the antibiogram of a sample using gnomonicus

Our translation of the WHOv2 catalogue [27], along with the variant call file produced by Clockwork and version 3 of the reference H37Rv genome (as a GenBank file), is then ingested by gnomonicus (v3.0.10) [26], which produces lists of the genetic variants detected in the sample (translated into amino acid mutations where appropriate), their predicted effects on antibiotics as specified by the catalogue, and finally the resulting predicted antibiogram. The resulting JavaScript Object Notation (JSON) files, along with the command used to generate them, are stored in the attendant repository [1]. Since the pipeline deployed in GPAS also uses gnomonicus, we also downloaded an output file for each sample using the same CLI that handled uploading; this file contains general information about the sample. All 5,326 output files are stored in the attendant GitHub repository [1] as described in the Data Summary.

### Comparison with TB-Profiler

All 2,663 samples were also processed by TB-Profiler (v6.6.5) [9], an established bioinformatic tool. It uses GATK [28] for variant calling rather than Clockwork. TB-Profiler produces a JSON file containing a list of detected mutations and their predicted effects on specific antibiotics. It not only implements its own version of WHOv2 but also includes additional rules for identifying resistance derived from the scientific literature. Unlike our GARC grammar, it has no Fail rule and only classifies mutations if they are listed in its catalogue. Hence, mutations in resistance genes that are not in the catalogue will be predicted to be Susceptible. Five of the 2,663 samples failed to return a result, and these were excluded from the subsequent analysis. The resulting output JSON files for the remaining 2,658 samples are included in the attendant GitHub repository [1], as is code to aggregate all the results into data tables, thereby enabling direct comparison with the results of gnomonicus.

## Results

### Some antibiotics performed slightly better, and some slightly worse when we applied a Simple implementation of the WHOv2 catalogue

**Fig. 1.**
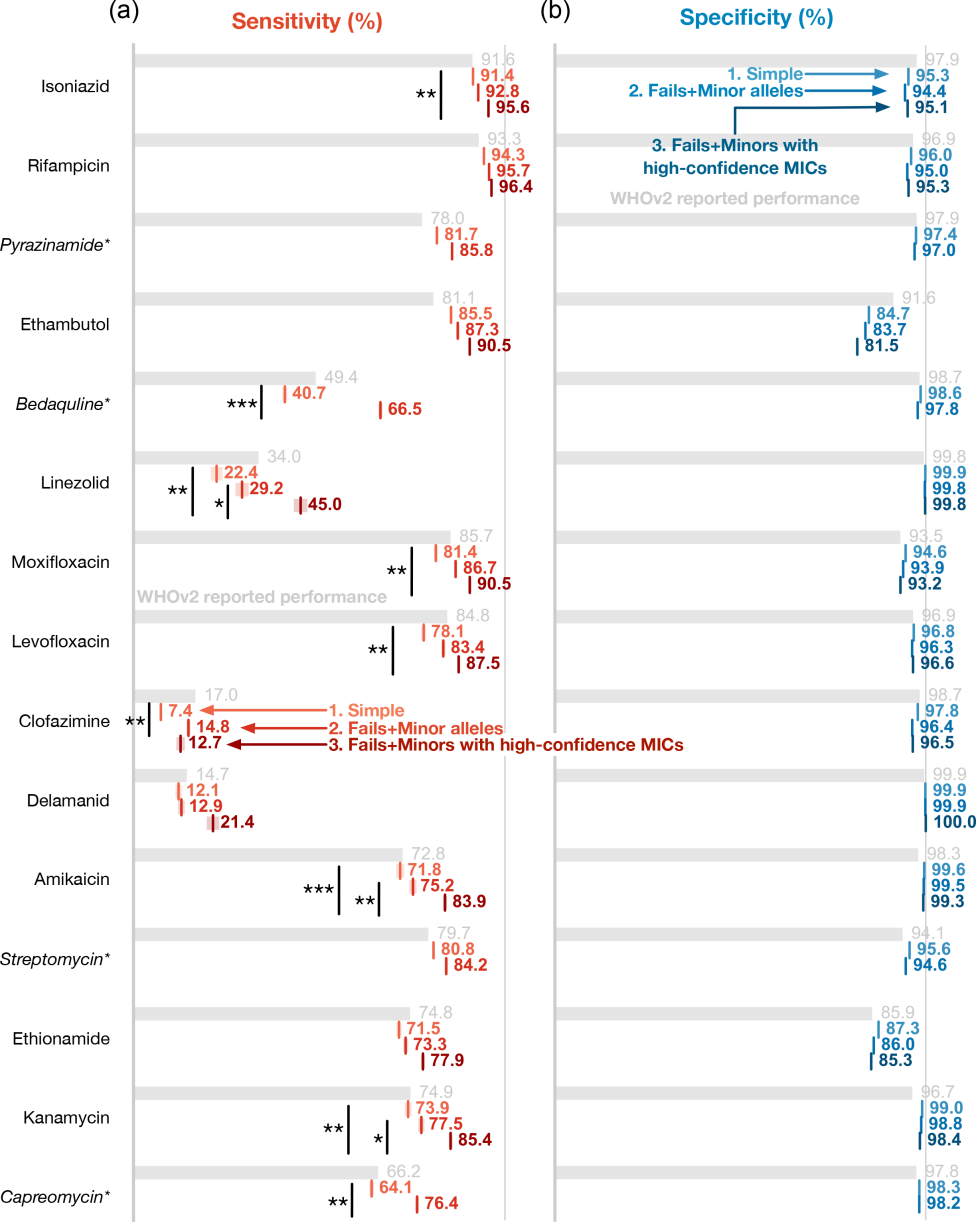
When tested on the 2,663 samples of the diverse test set, gnomonocius produces comparable antimicrobial resistance prediction performance using our translated version of the WHOv2 catalogue, as reported on a much larger dataset [27]. The (**a**) sensitivity and (**b**) susceptibility values for the 15 antibiotics included in the WHOv2 catalogue. Grey bars indicate performance reported on the WHO Training Dataset, and since these were calculated on a different, larger dataset, they are not directly comparable. Four drugs (marked with an asterisk) have phenotypes from MGIT testing. Our translation of the WHOv2 catalogue is applied in three different ways: (**i**) a straightforward application, (ii) allowing resistant minor alleles with three or more reads to be called and (iii) only comparing to high-confidence MICs in addition to calling minor alleles. Since the latter can only be calculated for antibiotics with UKMYC MICs, no values are reported for the four drugs measured by MGIT. Mantel–Haenszel statistics were calculated for all comparisons. Comparisons with *P*-values less than 0.05, 0.01 and 0.001 are annotated with one, two or three asterisks, respectively.

Since the WHOv2 catalogue does not provide any guidance on whether to detect minor alleles or what to do if there are insufficient reads at loci associated with resistance, we began by ignoring both of these effects and simply calculated the performance on our diverse test set – we call this the Simple implementation (Figs 1 and S1, available in the online Supplementary Material, and Tables 2 and S2). We found that five antibiotics had sensitivities within ±1.5% (rifampicin, isoniazid, streptomycin, amikacin and kanamycin; Fig. 1) of that reported by the WHO, two antibiotics (pyrazinamide and ethambutol) had higher sensitivities (>1.5%), and the remaining eight drugs (capreomycin, bedaquiline, linezolid, moxifloxacin, levofloxacin, clofazimine, delamanid and ethionamide) all recorded lower sensitivities (<1.5%) than seen on the WHOv2 Training Dataset. The variation in specificity was lower: two antibiotics (ethionamide and kanamycin) recorded higher specificities (>1.5%) than reported for WHOv2, with one drug (ethambutol) lower. Comparing our results to those published by WHOv2 on its own Training Dataset [8] is unfair, since the datasets are different, but at least gives us some idea of the performance, although we cannot conclude what is responsible for the observed differences.

**Table 2.** Performance of the WHOv2 catalogue on the diverse test set of 2,663 *M. tuberculosis* samples Fails+Minors includes rules that will Fail a drug if there are two or fewer reads at a locus associated with resistance, but will call Resistant if there are three or more reads at the same locus. Simple excludes these rules. Fails+Minors (high confidence) applies the same catalogue, but to a subset of the diverse test set with only MICs supported by two or more independent reading methods concur (high confidence). This is only possible for antibiotics with UKMYC pDST data – antibiotics marked with an asterisk used simple binary MGIT pDST and therefore have no value for this last category. Confidence limits were estimated by bootstrapping.

	Simple		Fails+Minors		Fails+Minor (high confidence)	
	Sensitivity	Specificity	Sensitivity	Specificity	Sensitivity	Specificity
Isoniazid	91.4±0.5	95.3±0.4	92.8±0.5	94.4±0.4	95.6±0.4	95.1±0.4
Rifampicin	94.3±0.5	96.0±0.3	95.7±0.4	95.0±0.4	96.4±0.3	95.3±0.4
Pyrazinamide*	81.7±0.7	97.4±0.3	85.8±0.7	97.0±0.4		
Ethambutol	85.5±0.8	84.7±0.6	87.3±0.8	83.7±0.6	90.5±0.6	81.5±0.6
Bedaquiline*	40.7±1.0	98.6±0.2	66.5±0.9	97.8±0.3		
Linezolid	22.4±1.9	99.9±0.1	29.2±2.1	99.8±0.1	45.0±2.2	99.8±0.1
Moxifloxacin	81.4±0.8	94.6±0.3	86.7±0.7	93.9±0.3	90.5±0.7	93.2±0.4
Levofloxacin	78.1±1.0	96.8±0.3	83.4±0.9	96.3±0.3	87.5±0.7	96.6±0.3
Clofazimine	7.4±0.7	97.8±0.2	14.8±0.9	96.4±0.3	12.7±1.5	96.5±0.2
Delamanid	12.1±1.2	99.9±0.2	12.9±1.2	99.0±0.1	21.4±2.0	100.0±0.0
Amikacin	71.8±1.4	99.6±0.1	75.2±1.3	99.0±0.1	83.9±0.9	99.3±0.1
Streptomycin*	80.0±0.7	99.6±0.3	84.3±0.8	99.4±0.6		
Ethionamide	71.5±1.0	87.3±0.5	73.3±1.0	86.0±0.5	77.9±0.9	85.3±0.5
Kanamycin	73.9±1.3	99.0±0.3	77.5±1.2	98.0±0.3	85.4±0.8	98.4±0.1
Capreomycin*	64.1±1.0	98.3±0.2	76.4±0.8	98.2±0.2		

### Failing samples with insufficient reads at loci associated with resistance is worthwhile but does not improve performance

If there are insufficient reads at a genetic locus, one does not know if the nucleotide is the same as the reference, is different, resulting in a SNP, or has even been deleted, perhaps leading to a frameshift. If the genetic locus is also one associated with resistance, then the likelihood is such that, even in the absence of genetic information, the sample has a reasonable probability of being resistant to the relevant antibiotic. This is a qualitatively different outcome from R, S or U, since re-sequencing is likely to result in more reads at that position, allowing a definite result to be returned. In our implementation of the WHOv2 catalogue, any genetic locus in a sample which has two or fewer (including zero) reads but could be a RAV was described as a Fail (F).

Adding these rules yielded the Fails implementation. Considering the genetic-based predictions for all 15 drugs using all 2,663 samples yielded 194 Fails from 39 genetic loci, which affected the antibiogram of 66 (2.5%) samples. The majority of Fail calls occurred in the *rrs* gene (106) followed by *rpoB* (20), *katG* (17), *pncA* (8) and *embB* (9). The resulting changes to the performance of streptomycin, rifampicin, isoniazid, pyrazinamide and ethambutol were small and lay within error and are therefore not shown. Note that the majority of loci in *rrs* with Fails did not contribute to amikacin and kanamycin resistance (according to WHOv2) and, therefore these antibiotics are, perhaps surprisingly, little affected.

### Identifying minor alleles containing RAVs improved performance

Thus far, all genetic variants have been identified using the default filter in the Clockwork pipeline, which requires 90% of reads to support a variant for it to be called. Note that this threshold is the same as that used in WHOv1 [7] but higher than the 75% used in WHOv2 [8]. Next, we added rules to our implementation of the WHOv2 catalogue such that any RAV supported by three or more short reads, regardless of how many reads are in support of other (usually WT) alleles, was called Resistant: this is the Fails+Minors implementation. The sensitivity of every antibiotic increased (Fig. 1, Table 2), with values ranging from +0.8% (streptomycin) to +9.9% (capreomycin). Nine antibiotics experienced an increase in sensitivity of 2% or more (pyrazinamide +4.1%, bedaquiline +25.8%, linezolid +6.8%, moxifloxacin +5.3%, levofloxacin +5.3%, clofazimine +7.4%, amikacin +3.4%, kanamycin +3.6% and capreomycin +12.3%), although only the increases seen for bedaquiline, clofazimine and capreomycin were statistically significant. Only clofazimine and ethionamide experienced reductions in specificity of more than 1% (1.4 and 1.3%, respectively); neither was statistically significant.

### Only comparing with high-confidence pDST results improves the measured performance

There are many reasons why we cannot perfectly predict the antibiogram for each sample; these include sample mislabelling, not all genes or genetic variants having been classified, pDST measurement errors and genetics not being a perfect predictor of phenotype. We are fortunate in that the UKMYC dataset of 1,000 samples has images of all the 96-well plates from when they were read by the laboratory scientist after 2 weeks’ incubation. As mentioned in the Methods, this allowed the CRyPTIC project to produce high-confidence MICs where two or more independent methods agreed on the value [6,11], thereby reducing measurement error. We will therefore examine the effect of only using these high-confidence MICs.

The sensitivities of ten of the 11 antibiotics for which we have UKMYC data increased (clofazimine being the exception) compared with the Fails+Minors dataset, with increases ranging from +1.4% for rifampicin to +15.8% for linezolid (Fig. 1, Table 2). Specificity was largely unchanged, with only ethambutol (−2.2%) changing by more than an absolute percentage point. Only the increases for linezolid, amikacin and kanamycin were statistically significant; if we compare back to the Simple dataset, then the increases for isoniazid, linezolid, moxifloxacin, levofloxacin, amikacin and kanamycin were statistically significant. No changes in specificity were statistically significant. This suggests that measurement error is partly constraining the measured performance of the WHOv2 catalogue. Notably, both rifampicin and isoniazid now achieve sensitivities and specificities above 95%, which is a requirement to pass ISO 20776-2 : 2021 [29].

### Discrepant samples do not have lower growth but, for some drugs, the MICs lie near the ECOFF/ECV

Our starting hypothesis for why the genetics and pDST results remained different was that the discrepant samples were more likely to have poor bacterial growth on the UKMYC plates after 2 weeks’ incubation, as this could lead to measurement error. Examining the distributions of bacterial growth (averaged from the two positive control wells) showed that this is not true (Figs S2 and S3) as, whilst there was large variation between samples, there was no significant difference in the distributions between, for example, the true positives and false positives. Whilst the original measurement was an MIC, this was binarized using an ECOFF/ECV [11], and hence our next hypothesis was that discrepants could arise if their MICs were close to the ECOFF/ECV, again leading to measurement error. Results varied by drug (Figs S2 and S3), but this appears at least partly true for ethambutol, moxifloxacin and delamanid. It is clear that the less bimodal the MIC distribution, the more likely this effect will lead to misclassification and thereby discrepants.

### The performance of gnomonicus is similar to TB-Profiler

**Fig. 2.**
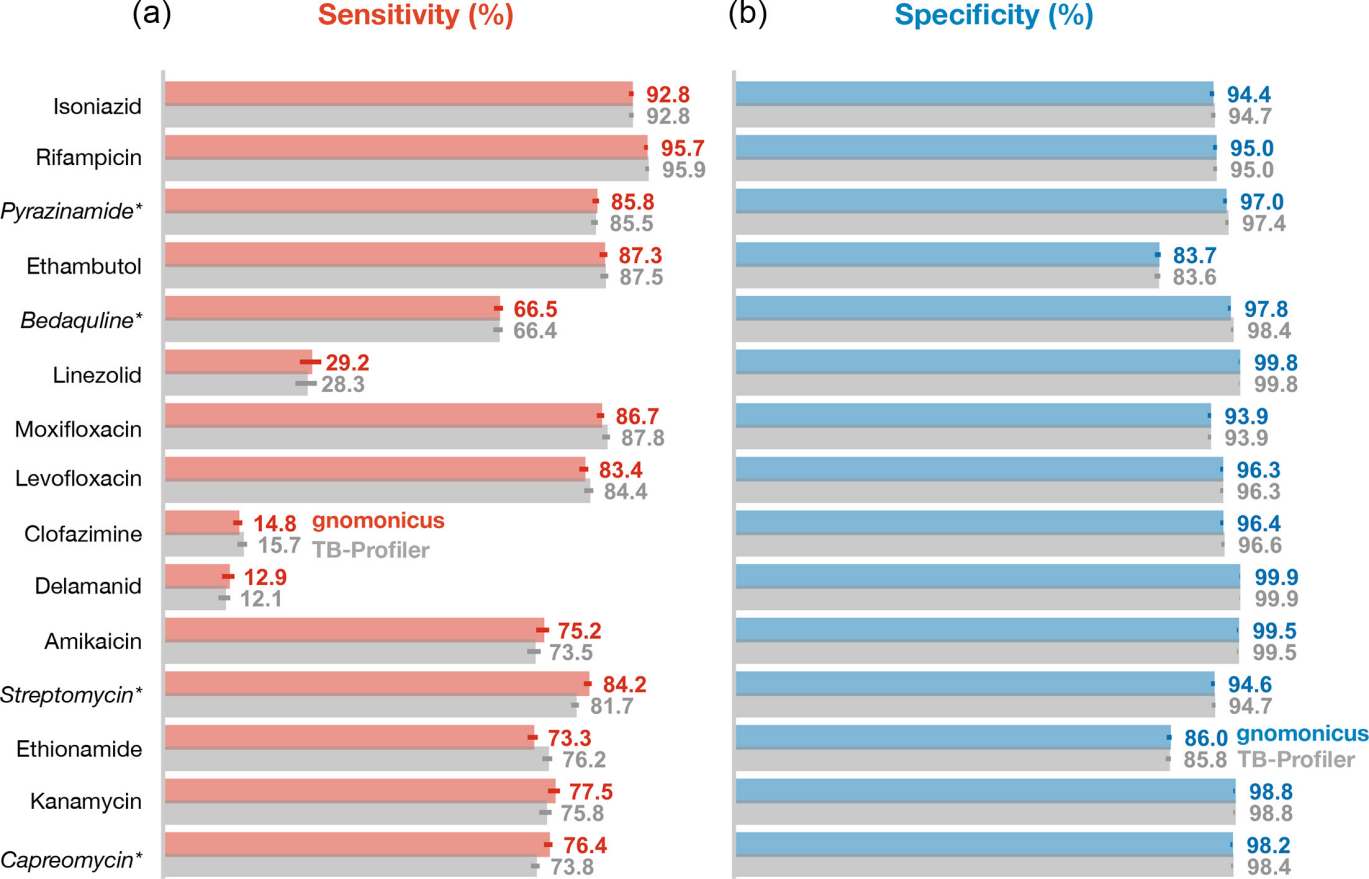
The performances of gnomonicus and TB-Profiler, measured using the 2,663 samples of the diverse test set, are very similar. The (**a**) sensitivities and (**b**) specificities for all the drugs in the WHOv2 are reported. To enable comparison for all drugs, the gnomonicus results are from the Fail +Minors implementation. Four drugs (marked with an asterisk) have phenotypes from MGIT testing. Applying a McNemar test to the paired results suggests that none of the differences reach significance (*P<*0*.*05).

The diverse test set of 2,663 samples was also run through TB-Profiler (Methods). The predicted effects of an antibiotic on a sample were compared where (i) both methods returned a result for a sample and (ii) there was also a matching pDST result, as per Table 1. There were no significant differences between the sensitivities and specificities calculated using both gnomonicus and TB-Profiler (Fig. 2); however, this approach is somewhat crude as it, for example, groups together Susceptible and Unknown predictions and does not consider differences in predictions at the level of an individual sample. Hence, let us drill down into the differences between the two tools.

Two methodological differences become apparent when comparing the predictions in aggregate across all drugs (Table 3): first, only gnomonicus returns a result of Fail (*n*=27), and second, gnomonicus returns nine times more Unknown results (*n*=1,071 versus 115). The former classification is unique to gnomonicus, whilst the latter is due to our decision to return a result of Unknown for mutations in resistance genes that are not explicitly listed in WHOv2. In these cases, TB-Profiler would instead return a result of Susceptible. The aim of both classifications is to avoid very major errors (VMEs); these are defined as resistant samples incorrectly predicted as susceptible [30]. The tools gnomonicus and TB-Profiler make 881 and 1,096 VMEs, respectively. The Fail category avoids five VMEs in this dataset, whilst the additional Unknown calls made by gnomonicus account for 205. Both tools (and thence the WHOv2 catalogue) have an aggregate VME rate far in excess of the 3% required by the Clinical and Laboratory Standards Institute (or the 1.5% mandated by the Food and Drug Administration), but this is mainly driven by the poor performance of a few drugs (Fig. 2, Table S3).

**Table 3.** Majority of classifications made by gnomonicus and TB-Profiler agree (12,729/13,821, 92.1%) Most of the differences (965/13,821, 7.0%) occur when gnomonicus returns an Unknown result due to a mutation in a resistance gene that is not listed in WHOv2, whilst TB-Profiler returns Susceptible. A minority of classifications (63/13,821, 0.46%) are true discrepants in the sense that one method returns Resistant and the other Susceptible. The number of classifications that are measured as resistant by a pDST method is given in parentheses. Table S3 contains a detailed breakdown by drug.

		TB-Profiler			
		Resistant	Susceptible	Unknown	Total
Gnomonicus	Resistant	3,705 (3,297)	43 (33)	0 (0)	3,748 (3,330)
	Susceptible	20 (18)	8,932 (853)	23 (10)	8,975 (881)
	Unknown	14 (11)	965 (205)	92 (33)	1,071 (249)
	Fail	4 (3)	23 (5)	0 (0)	27 (8)
	Total	3,743 (3,329)	9,963 (1,096)	115 (43)	13,821 (4,468)

Overall, both tools agree on a classification of Resistant, Susceptible or Unknown in 92.1% (12,729/13,821) of cases. As noted above, the majority of the difference (7.0%, 965/13,821) is due to our decision to return a result of Unknown for mutations in resistance genes not listed in WHOv2. That leaves 127 discrepants (0.92%), about half (64) of which should not lead to clinical error as one or other of the tools is returning a result of Unknown or Fail, which should, in practice, trigger additional investigations or tests. That leaves 63 (0.46%) predictions as true discrepants where one method has returned a result of Resistant and the other Susceptible.

The discrepants split into two groups; in 43 samples, gnomonicus predicts resistance whilst TB-Profiler does not, and in the remaining 20 samples, the situation is reversed, with the latter group being more enriched for resistance (18/20, 90.0% versus 33/43, 76.7%). Of these 63 predictions, gnomonicus and TB-Profiler are therefore correct in 33+2=35 cases and 18+10=28 cases, respectively, and hence, the behaviour of both tools is not significantly different in this dataset (McNemar test; *P*=0.45). Not all of these predictions are independent, as there are several members of the fluoroquinolones and aminoglycosides in WHOv2 which all have similar resistance mechanisms. Only considering a single member from each drug class does not alter the result.

Breaking down the 63 discrepants by drug shows that all drugs have between one (rifampicin, linezolid and delamanid) and nine (capreomycin) discrepant predictions (Table 4). All the genetic variants driving these 63 discrepant predictions are given in the Supplement (Table S4). The main conclusion is that the discrepants are mainly due to differences in the upstream genetic-processing pipelines detecting different genetic variants in a small number of samples, rather than differences in how the WHOv2 catalogue has been translated and incorporated into both tools. This is encouraging but illustrates the importance of the upstream variant caller. The only cases where a variant is detected by both tools but only one predicts resistance are those in which there is evidence of two SNPs at a single locus in a resistance gene, here *gyrA* (three samples, Table S4) and *katG* (one sample). TB-Profiler identifies the presence of two mutations (e.g. D94N and D94Y in *gyrA*) and, since both are associated with resistance to levofloxacin and moxifloxacin, predicts resistance to both drugs. Since all seven cases are phenotypically resistant, TB-Profiler is correct. By contrast, gnomonicus notes that there is a heterogeneous call but does not resolve it further and therefore no catalogue rules are triggered, and hence a result of susceptible is returned which is incorrect. This accounts for seven of the 20 cases where TB-Profiler calls resistance and gnomonicus does not (Table S4). Five of the remaining 13 cases are due to TB-Profiler detecting a single-base deletion in *Rv0678/mmpR5* at position 198.

**Table 4.** Sixty-three discrepant classifications produced by gnomonicus and TB-Profiler, broken down by drug The quadrants from the truth table are listed with the gnomonicus result first and the TB-Profiler result second e.g. RS = gnomonicus predicted resistant, TB-Profiler predicted susceptible. The numbers of paired discrepants are too small to support useful statistical testing.

Drug	RR	RS	SR	SS
Isoniazid	483	2	1	467
Rifampicin	480	0	1	497
Pyrazinamide	256	3	0	351
Ethambutol	360	0	2	576
Bedaquiline	268	5	3	456
Linezolid	34	1	0	882
Moxifloxacin	290	1	4	632
Levofloxacin	290	1	4	636
Clofazimine	64	2	3	878
Delamanid	18	1	0	739
Amikacin	213	5	0	719
Streptomycin	224	6	0	313
Ethionamide	304	4	0	498
Kanamycin	226	5	0	704
Capreomycin	195	7	2	584
Total	3,705	43	20	8,932

Twenty-two of the 43 cases where gnomonicus predicts resistance are due to variants identified in the *rrs* gene. Only 17 samples are affected because mutations in this gene are implicated in aminoglycoside resistance, but of these, nine are due to the presence of a1401g at varying levels of support, which is not detected and therefore called by TB-Profiler. Since 15 out of 16 of the resulting predictions are phenotypically resistant, we infer that gnomonicus is correct in these cases. Another five cases are due to other variants detected in *rrs* by gnomonicus and, interestingly, four cases are due to the identification of large deletions in *ddn* (22 bases), *Rv0678* (76 bases), *gid* (133 bases) and *ethA* (700 bases), leading to frameshifts and/or loss of function that are not picked up by TB-Profiler.

## Discussion

We have characterized the performance of our antimicrobial resistance prediction tool, gnomonicus, using the second edition of the WHO catalogue of resistance-associated mutations [8] and compared its performance to that of TB-Profiler [9], an established bioinformatic tool with similar functionality. Whilst WHOv2 is an accepted and well-known catalogue, we have had to, in effect, translate the catalogue, since the published catalogue is not computer-parsable, and therefore we are testing two distinct statements: (i) that our translation is correct and (ii) that the reported performance for WHOv2 [8] is representative of what would be expected in the clinic. Unfortunately, the samples that make up the WHOv2 Training Dataset are not (yet) publicly available, which would have helped us with the first point. There are likely very good reasons for this, for example, data owners may be willing to share samples with the WHO but not the public, but it does prevent researchers, such as ourselves, from reproducing the analysis that led to WHOv2, which is important to identify errors and gain trust. We cannot therefore formally disentangle whether the observed differences are due to differences between the WHO Training Dataset and our publicly available diverse test set, errors we have made parsing the catalogue, or assembly and variant calling differences. Some reassurance is gained on both points from gnomonicus (i) achieving similar performance for many of the 15 antibiotics covered by WHOv2 using our diverse test set of 2,663 *M. tuberculosis* samples and (ii) that its results are in close agreement with those produced by TB-Profiler [9]. If we only compare our results with high-confidence pDST results, the two main antitubercular drugs, rifampicin and isoniazid, achieve sensitivities and specificities above 95%, which is a requirement of ISO 20776-2 : 2021 [29].

Since there is no published guidance on how to *apply* WHOv2 to a WGS sample, we have chosen to make three enhancements; the first is to explicitly flag genetic loci that are associated with resistance when there are insufficient reads to identify the allele. These we call Fails and, whilst small in number (2.5% of samples had at least one Fail), they correlate with poor sequencing quality and usually indicate a sample needs to be re-sequenced. The second improvement is that we have chosen to classify mutations in genes known to be associated with resistance as Unknown, even if they are not explicitly listed in the WHOv2 catalogue. This is possible because our GARC grammar (defined in the Supplement) allows wildcards such that our catalogues can contain a single rule encoding logic like ‘any missense mutation in the coding region of gene X’, which, in addition to a way of prioritizing rules, is necessary to enable this functionality. Finally, gnomonicus identifies any RAV if it is supported by at least three (short) reads, thereby allowing minor alleles to contribute to resistance prediction. This has been shown to boost the sensitivity for fluoroquinolones [31], rifampicin [32] and bedaquiline [33], and the WHOv2 report also showed how lowering the fraction of reads supporting a variant call often increased the sensitivity [8]. The net effect of these changes was that nine antibiotics saw an increase in sensitivity of more than two absolute percentage points, but this was only statistically significant for bedaquiline, clofazimine and capreomycin; a larger dataset is needed to draw definite conclusions. Only two drugs saw a drop in specificity of more than one absolute percentage point, and neither decrease was statistically significant. The effect of these enhancements can also be clearly seen when the results of gnomonicus and TB-Profiler are compared; the latter only implements the last enhancement and therefore does not return a result of Fail, and gnomonicus returns almost an order of magnitude more Unknown results due to the second enhancement, both of which help reduce the number of VMEs.

To calculate the sensitivity and specificity, one must have a binary outcome, yet the WHOv2 catalogue yields three: Resistant, Susceptible and Unknown. This was achieved in the WHOv2 report by assuming all samples classified as Unknown were susceptible. So that we may compare our results with those reported for WHOv2 [8], we have done the same (Figs 1 and 2, Table 2). As we have noted, gnomonicus returns more Unknown classifications than TB-Profiler, mainly because it detects and calls any SNP, insertion or deletion in a resistance gene that is not explicitly listed in WHOv2; combining Susceptible and Unknown results therefore masks this difference. Aggregating Susceptible and Unknown results like this is reasonable for antibiotics where the majority of the genetic determinants have been discovered, since the conditional probability of a novel variant conferring resistance is low, but for drugs like bedaquiline or even pyrazinamide, this assumption breaks down. For these drugs, at least, it seems sensible to report an Unknown result for the drug, rather than assume it is Susceptible. Other studies have shown how, in some cases, one can take advantage of the correlations between different drugs to infer that they are likely Susceptible [34].

Most catalogues of RAVs and bioinformatic tools, such as TB-Profiler, use a three-valued logic: Resistant, Susceptible and Unknown. Here we introduce a fourth value, Fail, that is different from the others, since it indicates we are lacking information at one or more genetic loci associated with resistance; in practice, this usually means the sequencing was of poor quality and the run needs to be repeated. It is worth reflecting, however, on the Unknown value, as this is, in fact, composed of two distinct cases. These are delineated by whether the genetic variant has been seen in sufficient clinical samples to have adequate statistical support or not. If not, then the label is transitory; collecting more samples will improve the statistics, and its effect will become associated with a definite label – we suggest this retains the Unknown label. But if sufficient samples are present in the Training Dataset, then it corresponds to genetic variants, such as M306V/I in *embB*, where the MIC distribution straddles the ECOFF/ECV. Collecting more samples changes nothing, and one can argue that another *definite* value, distinct from Resistant or Susceptible, is therefore needed. Naming such a value is controversial (e.g. Intermediate), but its existence is unavoidable and undeniable.

Clearly, it would have been preferable to be able to isolate our implementation of the WHOv2 catalogue in our Mycobacterial pipeline from the performance of the WHOv2 catalogue, but this was not possible due to the lack of publicly available datasets. Also, whilst we have described our 2,663 samples as a diverse test set, it is likely not truly independent, since at least the 1,000 UKMYC samples form part of the WHO Training Dataset. Other shortcomings of our diverse test set include that (i) we were unable to obtain, for all drugs, a 50 : 50 split in resistance/susceptibility, which reduces our statistical resolving power for those drugs and (ii) that it is not designed to be genetically diverse, so does not test all rules in a catalogue. Finally, despite our best efforts, there are insufficient samples resistant to bedaquiline, linezolid and delamanid in the diverse test set; additional, targeted collection of samples, which are then made publicly available, is the only answer here.

Our aspiration is that open-source tools, like gnomonicus, will help accelerate the uptake of WGS by public health bodies for TB surveillance and diagnostics [35]. To characterize its performance, we have constructed and made available a diverse test set of 2,663 *M. tuberculosis* samples; we hope this will be of use to other researchers and could become a standard dataset upon which new tools and/or catalogues could be tested, allowing the consistent reporting of preliminary performance. All samples and their results can be downloaded using the attendant GitHub repository [1], which also contains code allowing all analysis to be repeated and all figures to be redrawn.

## Supplementary material

10.1099/mgen.0.001592 Uncited Fig. S1.

## Abbreviations

AMR: antimicrobial resistance
BMD: broth microdilution
BPaLM: bedaquiline, pretonamid, linezolid and moxifloxacin
CLI: command-line interface
CRyPTIC: Comprehensive Resistance Prediction for Tuberculosis: an International Consortium
ECOFF/ECVs: epidemiological cutoff values
EIT: Ellison Institute of Technology
ENA: European Nucleotide Archive
GARC: Grammar for Antimicrobial Resistance Catalogues
GPAS: Global Pathogen Analysis Service
HGVS: Human Genome Variation Society
JSON: JavaScript Object Notation
MDR: multi-drug-resistant
MGIT: Microbial Growth Indicator Tube
MIC: minimum inhibitory concentration
pDST: phenotypic drug susceptibility testing
RAV: resistance-associated variant
TB: tuberculosis
VCF: variant call format
VME: very major error
WGS: whole-genome sequencing
WHO: World Health Organization
